# Myrrh Oleo‐Gum Resin as a Functional Additive in Pectin and κ‐Carrageenan Composite Films for Food Packaging

**DOI:** 10.1002/fsn3.4524

**Published:** 2024-11-05

**Authors:** Yasir Abbas Shah, Saurabh Bhatia, Sampath Chinnam, Ahmed Al‐Harrasi, Mohammad Tarahi, Talha Shireen Khan, Tanveer Alam, Esra Koca, Levent Yurdaer Aydemir, Anil K. Philip, Muhammad Afzaal, Mahbubur Rahman Khan, Anubhav Pratap‐Singh

**Affiliations:** ^1^ Natural and Medical Sciences Research Center University of Nizwa Nizwa Oman; ^2^ School of Health Sciences School of Health Science, University of Petroleum and Energy Studies Dehradun India; ^3^ Department of Chemistry M.S. Ramaiah Institute of Technology Bengaluru Karnataka India; ^4^ Department of Food Science and Technology School of Agriculture, Shiraz University Shiraz Iran; ^5^ Nanotechnology Research and Application Center Sabanci University Nanotechnology Research and Application Center, Sabanci University, Orta Mahalle Istanbul Turkey; ^6^ Department of Food Engineering, Faculty of Engineering Adana Alparslan Turkes Science and Technology University Adana Turkey; ^7^ School of Pharmacy School of Pharmacy, University of Nizwa Nizwa Oman; ^8^ Food Safety and Biotechnology Lab, Department of Food Science Government College University Faisalabad Faisalabad Pakistan; ^9^ Department of Food Processing and Preservation Hajee Mohammad Danesh Science and Technology University Dinajpur Bangladesh; ^10^ BC Food and Beverage Innovation Centre, Faculty of Land and Food Systems The University of British Columbia Vancouver British Columbia Canada

**Keywords:** antioxidant, biopolymer films, food packaging, oleo‐gum resin

## Abstract

Myrrh oleo‐gum‐resin (MOGR) is a natural substance that has a rich history of medicinal use due to its anti‐inflammatory, antimicrobial, and antioxidant properties. The present study reports on the fabrication and assessment of pectin and K‐carrageenan composite films infused with varying proportions (0.3%, 0.5%, and 0.7%) of MOGR. Morphological analysis of the film samples was conducted using Scanning Electron Microscopy (SEM) and Atomic Force Microscopy (AFM). The results indicated that the introduction of MOGR led to a notable increase in surface roughness. The SEM micrographs of the films showed that the MOGR addition had an important effect on the microstructure of the film. The surface hydrophobicity of the MOGR‐loaded films increased, as confirmed by the rise in the contact angle. Moreover, there was an increase in the thickness (0.062 ± 0.004–0.095 ± 0.006 mm) and opacity (1.24 ± 0.07–9.41 ± 0.24) of the films with the addition of MOGR; however, tensile strength (7.30 ± 0.50–4.92 ± 0.34 MPa), elongation at break (32.41% ± 1.0%–29.70% ± 0.24%), and barrier properties decreased. Additionally, a rise in MOGR concentration corresponded to a rise in overall color difference Δ*E* (0.77 ± 0.03–5.09 ± 0.49) of the films. Notably, the incorporation of MOGR led to an increase in the antioxidant activity of the composite films, indicating potential applications in functional packaging materials.

## Graphical abstract

Visual examination of the prepared film samples based on pectin and κ‐carrageenan composite loaded with myrrh oleo‐gum resin as a functional additive.

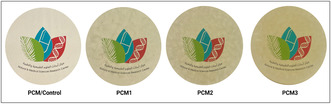

## 1 Introduction

The production of large amounts of nonbiodegradable plastics has put tremendous pressure on the world's natural environment, as it is estimated that only 14% of plastic waste is recycled worldwide (Aga et al. 2021; Zhang, Hadidi, et al. 2023). Therefore, to reduce environmental pollution and meet the consumer demand for the application of biodegradable and sustainable materials in food packaging, the development of polysaccharide‐based films has gained a lot of attention in recent years. However, these biopolymers have some disadvantages, for example, poor mechanical and vapor barrier properties, that restrict their further food packaging applications (Zhang, Hadidi, et al. 2023; Marangoni Júnior et al. 2022). As a result, several techniques, such as mixing two or more distinct biopolymers, have been developed to enhance these techno‐functional features of the polysaccharide‐based films.

Pectin is a heteropolysaccharide that has been used in the preparation of edible films. It shows a low tensile strength (TS) and a quite brittle structure, which limits their industrial applications (Chandel et al. 2022; Huang et al. 2021). On the other hand, carrageenan is a biopolymer that can be extracted from different species of red seaweed. Due to the excellent hydrophilic properties of κ‐carrageenan along with the high content of sulfate ester groups (30%–25%), it can be regarded as an appropriate biodegradable substance for use in food packaging (Candogan and Kolsarici 2003; Popov et al. 2022; Günter et al. 2022). However, in the industry, the composite material consisting of two or more polymers is preferred as it allows to provide a wider range of mechanical and textural properties. Therefore, the combination of pectin and κ‐carrageenan can allow the obtaining of novel composite materials with favorable functionality in food packaging applications.

Since ancient times, medicinal plants have been used for many medicinal purposes and are widely known. *Commiphora myrrha* (Nees) Engl., called myrrh, belongs to the family Burseraceae, which is a native of desert regions of the Middle East and Northern Africa. Traditionally, the resins and oils of these trees were used in Ayurvedic, Siddha, and Unani medicines as antibacterial, antifungal, anti‐inflammatory, anticoagulant, anti‐diabetic, and anti‐obesity agents (Morikawa, Matsuda, and Yoshikawa 2017; Hassanzadeh‐Taheri et al. 2021). Myrrh oleo‐gum‐resin (MOGR) is a yellowish‐reddish resinous substance, extracted from the damaged bark of the *Commiphora* genus (Hosseinkhani et al. 2017). Previous studies have shown the inhibitory activities of MOGR against the growth of several pathogenic bacteria. It also contains eugenols and aldehydes that are oxygen radical scavengers with anti‐lipemic and anti‐mutagenic potentials (Alshibly et al. 2022; Perveen et al. 2018). These results indicate MOGR as a biocompatible, low‐cost, and safe antimicrobial/antioxidant agent that can be used in food packaging.

There are limited published articles on the fabrication of active films by the incorporation of MOGR. Hence, the objective of the current study is to investigate the possible preparation of novel films using two different types of polysaccharides including pectin and κ‐carrageenan, as well as MOGR, as a natural bioactive compound.

## 2 Materials and Methods

### 2.1 Materials

Pectin, κ‐carrageenan, and tween 80 were purchased from Sisco Research Laboratories Pvt. Ltd. (SRL), India. Glycerol (99.0% purity) was provided from BDH Laboratory Supplies, UK. Myrrh resin was received as a gift sample from Fine Aromatics and Herbal Extract Pvt. Ltd. (a subsidiary of Fine Fragrances Pvt. Ltd.), Umbergaon, India. *Commiphora myrrha* gum was identified by Dr. B. S. Kalakoti (Taxonomist), Head R&D, Fine Aromatics and Herbal Extract Pvt. Ltd. Other chemicals used in this were purchased from Sigma‐Aldrich (St Louis, MO, USA) with an analytical grade.

### 2.2 Extraction of Myrrh Oleogum Resin (Absolute)

The powder resin (1.0 kg) was extracted with absolute ethanol (3.0 L) at 50°C with continuous stirring for 3 h (3 times). At the end of each extraction, the solvent was filtered to get a clear solution. Finally, all three extractions were mixed and distilled out by rotary evaporator under reduced pressure to get the thick sticky mass, that is, Myrrh gum oleoresin (250 g).

### 2.3 Edible Films Preparation

Pectin and k‐carrageenan composite films loaded with various concentrations of MOGR were prepared by the casting method (Bhatia et al. 2024). 2% (w/v) pectin and 1% (w/v) K‐carrageenan solutions were prepared separately by mixing the biopolymers with distilled water. The plasticizer (glycerol) and MOGR were added in different concentrations, as shown in Table 1. PCM film sample was kept at control, and PCM1‐PCM3 were added with different concentrations of MOGR. Twenty milliliters of the solution from each sample were transferred into the petri plates and subsequently shifted for drying.

**TABLE 1:** The compositional ratios of the components used for the film forming solutions.

Codes	Pectin (w/v)	κ‐Carrageenan (w/v)	Glycerol (v/v)	Myrrh oleo‐gum resin (w/v)
PCM/Control	2%	1%	1.5%	Blank
PCM1	2%	1%	1.5%	0.3%
PCM2	2%	1%	1.5%	0.5%
PCM3	2%	1%	1.5%	0.7%

### 2.4 Thickness

A Mitutoyo digital micrometer (2046F, Kawasaki, Japan) was used to determine the thickness of the film samples following established protocols. The average of 10 different positions was measured to obtain the average thickness of each film in millimeters (Bhatia, Shah, et al. 2023).

### 2.5 Barrier Properties

The WVP of the samples was measured using gravimetric methods with glass cups that had an inner diameter of 5 cm and a depth of 2 cm. The films underwent conditioning in a desiccator for 10 days at a relative humidity (RH) of 50%. To establish various RH conditions, silica gel (RH = 0%) and water (RH = 100%) were also employed. The calculation of WVP was carried out utilizing Equation (1).

(1) $$\mathrm{WVP}=\frac{∆m}{∆t\times ∆p\times A}\times d$$

In equation (1), “∆*m*/∆*t*” indicates the weight of the moisture gain per unit of time (g/d), “∆*P*” represents the water vapor pressure among the two sides of the film (kPa), “*A*” signifies the film area (m^2^), and “*d*” presents the film thickness in millimeters.

### 2.6 Transparency and Color Properties

The assessment of transparency and color attributes is pivotal for understanding the optical properties and visual appearance of materials such as edible films. Utilizing advanced instrumentation like spectrophotometers and chroma meters enables precise measurements, ensuring accuracy and reliability in the evaluation process. The degree of transparency exhibited by the films was evaluated utilizing an ONDA‐Vis spectrophotometer (Padova, Italy) set to a wavelength of 550 nm, following the methodology outlined by Zhao and Wang (Zhao, Wang, and Liu 2022). Furthermore, the color characteristics of the samples, including lightness (L^⁎^), redness (a^⁎^), and yellowness (b^⁎^), were analyzed using a CR‐410 Chroma Meter from Konica Minolta Sensing Americas (Tokyo, Japan). To quantify the overall color variation (Δ*E*) present in the films, Equation (2) was employed for evaluation.

(2) $$\Delta E={\left(∆L^{2}+∆a^{2}+∆b^{2}\right)}^{1/2}$$

In Equation (2), “Δ*L*,” “Δ*a*,” and “Δ*b*” represents the differences between the color parameters of the films and the white standards (L* = 95.9, a* = −0.09, b* = 0.1).

### 2.7 Mechanical Testing

This evaluation followed the guidelines outlined in the ASTM D882 standard method (ASTM 2010), a widely accepted protocol for such analyses, ensuring consistency and comparability of results. Before subjecting the samples to mechanical testing, it was imperative to standardize their environmental conditions to minimize external influences on the material properties. Therefore, the samples were carefully conditioned for an extended period of at least 40 h within a controlled test cabinet (Nüve TK 120, Türkiye), where specific parameters such as temperature (25°C) and relative humidity (50% RH) were maintained. Subsequently, the samples were precisely prepared into uniform strips (70 mm x 7 mm) and securely clamped between the grips of a texture analyzer equipped with 5 kg load cell (TA. XT plus from Stable Micro Systems). The acquired data were then analyzed using software, Exponent Connect, which facilitated the calculation and interpretation of the TS and EAB values.

### 2.8 Morphological Properties

According to the methodology described by Bhatia and Al‐Harrasi (Bhatia, al‐Harrasi, et al. 2023), the morphological characteristics of the film samples were examined using a scanning electron microscope (SEM, JSM‐6510 LA, Jeol, Japan) at an accelerating voltage of 15 kV.

### 2.9 Atomic Force Microscopy

The films were analyzed topographically using AFM technology (hpAFM, Nano‐Magnetics Instruments). Surface images were obtained by employing tapping mode scanning at room temperature. A specialized ACLA cantilever with a spring constant ranging from 36 to 90 N/m was utilized, and scan dimensions were set at 10 μm × 10 μm, with surface relief height recorded at a resolution of 256 pixels × 256 pixels. Three scans were performed for each sample at random positions on the thin film surface. Average roughness (Ra) and Root Mean Square roughness (Rq) values of the films were then determined based on the mean data plane.

### 2.10 XRD

The XRD examination was performed utilizing a Bruker D8 Discover apparatus operating at 40 kV. The film samples underwent scanning within the 2*θ* angle range, employing a scan rate of 0.500 s per data point, covering angles from 5° to 55°. This analytical technique allowed for the determination of the structural characteristics of the samples based on the diffraction patterns generated.

### 2.11 FTIR Spectroscopy

The FT‐IR spectra were obtained with an infrared Bruker Tensor 37 spectrometer (Ettlingen, Germany) at a resolution of 4 cm^−1^ in the range of 4000–400 cm^−1^.

### 2.12 Antioxidant Activity

The antioxidant activity of film samples was evaluated by both ABTS and DPPH assays, as described by Re, Pellegrini (Re et al. 1999), and Brand‐Williams, Cuvelier (Brand‐Williams, Cuvelier, and Berset 1995), respectively. For ABTS cation free radical scavenging assay, absorbance change at 734 nm was monitored for 6 min after mixing films with ABTS cation radical solution. The DPPH radical scavenging assay involved mixing films with DPPH radical solution and monitoring absorbance change at 517 nm after 30 min.

### 2.13 Statistical Analysis

The mean ± standard deviation (SD) of the triplicate results was utilized for statistical analysis. Using Duncan's test and SPSS software (V 17.0, IBM Company, Chicago, IL, USA), a one‐way analysis of variance (ANOVA) was performed between means at a predefined significance level of 5%.

## 3 Results and Discussion

### 3.1 Visual Analysis

**FIGURE 1:**
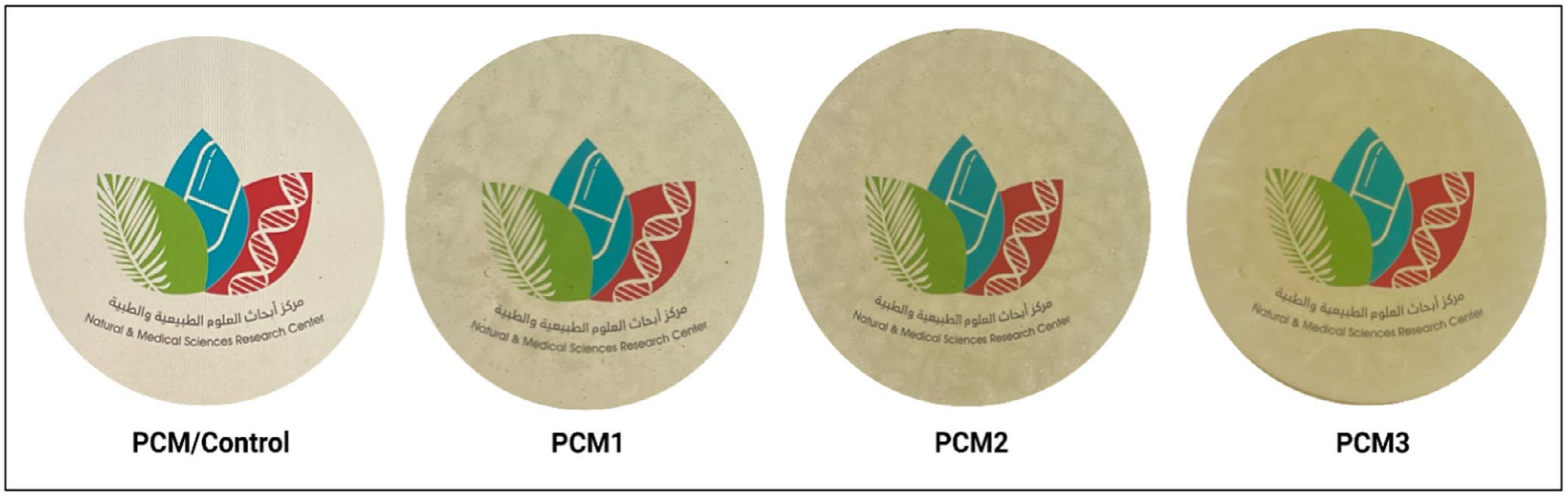
Visual examination of the prepared film samples.

Visual analysis is a crucial step in screening films based on their appearance, mechanical strength, and surface properties. Visual analysis is also important to check the presence of any polymeric aggregates or particles that may develop due to the improper homogenization or incompatibility of the additive with the film‐forming solution. Visual assessment of the blank and MOGR‐incorporated samples showed a decrease in transparency with an increase in the concentration of MOGR (Figure 1). Furthermore, surface roughness increased with an increase in the concentration of MOGR, as presented in Figure 1. However, there was no change in mechanical strength, such as the flexibility of the films.

### 3.2 Morphological Properties

#### 3.2.1 SEM Analysis

**FIGURE 2:**
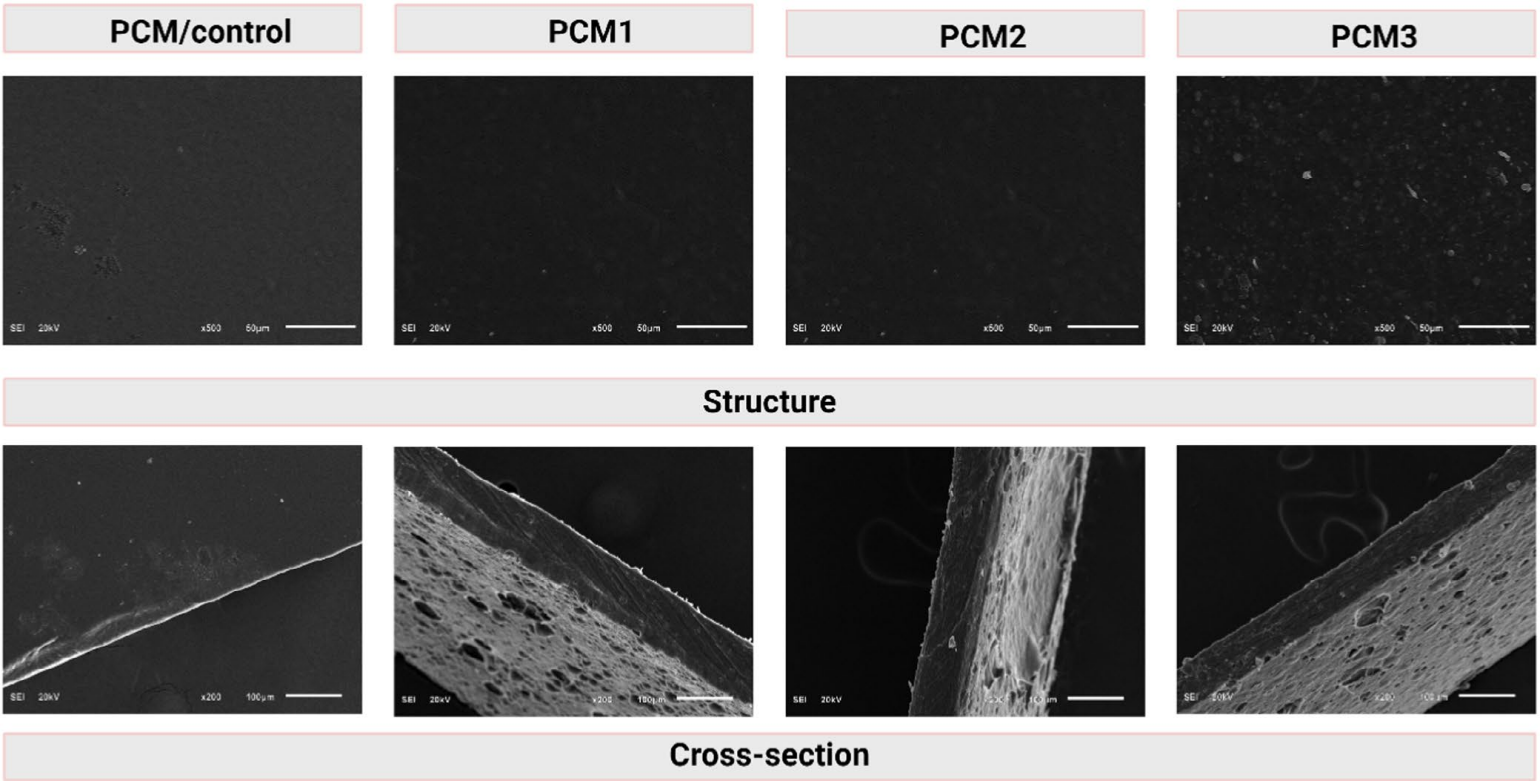
SEM micrographs of film samples (control) and loaded with MOGR.

**FIGURE 3:**
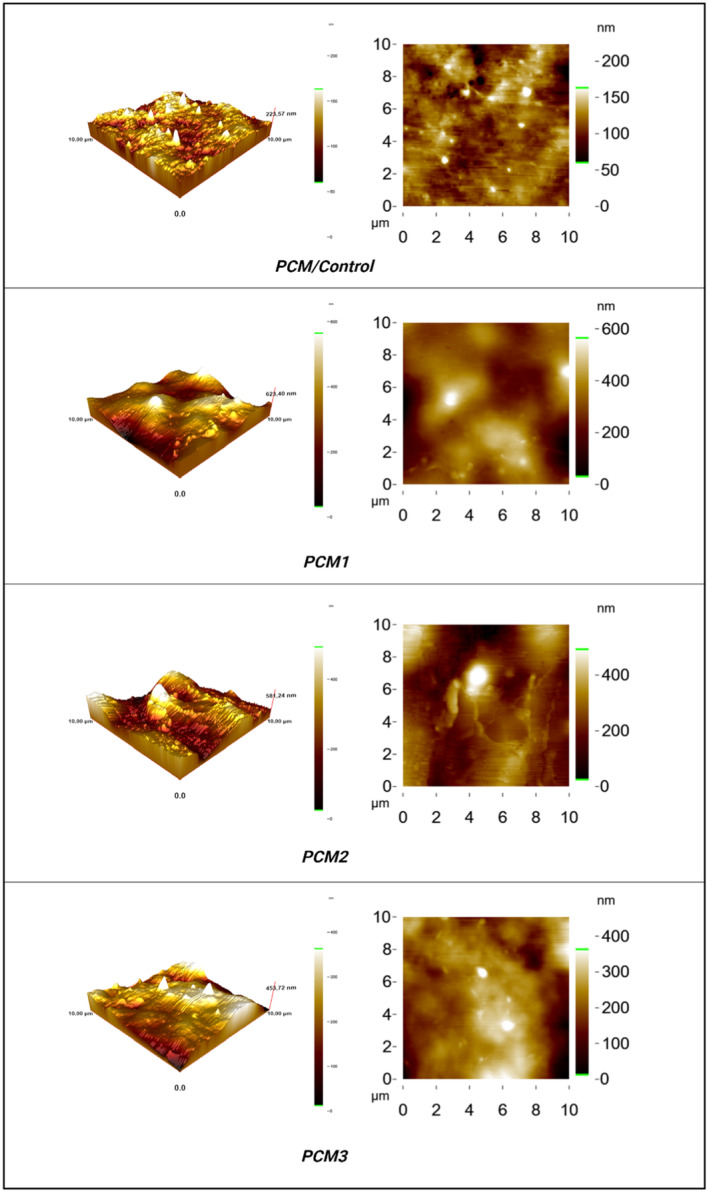
Morphological properties of the film samples including control and films loaded with MOGR.

SEM micrographs of the films (blank and MOGR incorporated) are shown in Figure 2. As observed in Figure 2, the microstructure of the control (PCM film) sample appeared smooth, compact, and uniform. The amount of MOGR had an important effect on homogeneous dispersibility in the film matrix. This outcome was consistent with the findings received from visual, and AFM results (Figures 1 and 3) and (Table 3). Several MOGR aggregates were observed in the films (PCM1‐PCM3). The micrographs also showed the appearance of pores in PCM1‐PCM3 films. Our findings are in accordance with the results obtained in our previous study, where the addition of *Boswellia sacra* oleo gum resin (ethanolic fraction) in biopolymeric films led to an increase in the surface roughness of the resultant films (Bhatia, Wasef, et al. 2023). Thus, based on the microstructures of PCM1‐PCM3 films, it can be concluded that the addition of MOGR caused aggregates and pores in the microstructure of the films.

#### 3.2.2 AFM Analysis

**FIGURE 4:**
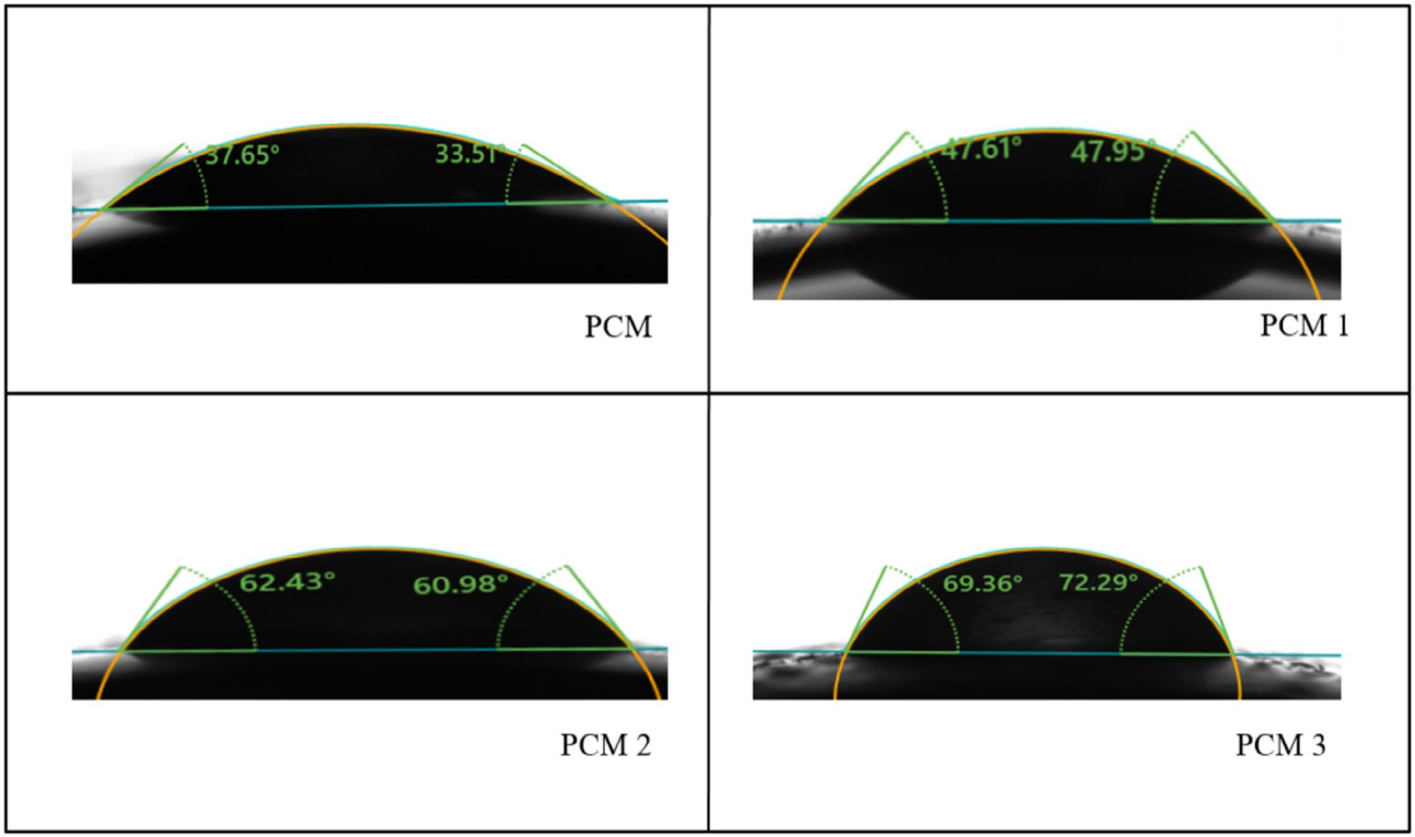
Contact angle of the film samples including control and films loaded with MOGR.

Atomic force micrographs presenting three‐dimensional images of the control (PCM) and the MOGR‐loaded films (PCM1, PCM2, and PCM3) are presented in Figure 4. The surface properties of all films examined by AFM showed almost similar roughness patterns as observed in SEM (Figure 2). Ra and Rq values of all film samples are presented in Table 2. The blank film (PCM) showed a smoother surface with Ra and Rq values of 12.63 and 16.56 nm, respectively, whereas the MOGR‐loaded film showed a rough surface, as designated by high values of Ra and Rq.

**TABLE 2:** The average roughness (*R*_a_) and the root‐mean‐square roughness (*R*_q_) values of the film samples.

Film samples	MOGR concentration (%)	*R*_a_ (nm)	*R*_q_ (nm)
PCM	Blank	12.63	16.56
PCM1	0.3	66.55	86.27
PCM2	0.5	63.07	81.09
PCM3	0.7	49.49	63.34

The intense peaks formed in MOGR‐loaded films could be attributed to the undissolved matter formed in the film‐forming solution after the addition of MOGR. The incorporation of MOGR augmented the surface roughness of the films, resulting in the formation of more bulges, as observed in Figure 3 (PCM1, PCM2, and PCM3). The blank film revealed a matrix that was relatively continuous, smooth, and free of pores and cracks. This increase in surface roughness could be linked to the aggregation of MOGR particles during the drying step, resulting in the development of irregularities on the film's surface (Ghasemlou et al. 2013). This could also be associated with the incompatibility of MOGR with film film‐forming solution resulting in the formation of irregularities on the film surface (Ghasemlou et al. 2013).

### 3.3 Contact Angle

The surface hydrophobic property of the films is a key characteristic that controls their sensitivity to moisture. Goniometers are frequently used to measure the contact angle that forms between a water droplet and the film surface to determine the hydrophobic properties of the film surface. The contact angle of the films represents the interaction between the film surface and solvent, known as the wettability of the films. The range of the contact angle is 0° (full dispersion of the liquid onto the solid surface) to 180° (the impracticable limit of no wetting) (Khazaei et al. 2014). The difference between a hydrophilic and hydrophobic surface is that the former has a water contact angle smaller than 90°, while the latter has one larger than 90° (Niu et al. 2019).

As shown in Figure 4, the contact angle increased in MOGR‐loaded films; it could be associated with the presence of hydrophobic essential oil and alcohol‐soluble resins in MOGR that raised the overall hydrophobicity of the film surface (Ajiteru et al. 2022). As per the previous finding, an increase in the contact angle could be attributed to rougher surfaces as a result of hydrophobic substance aggregation and then recrystallizing during the film‐drying process (Muscat et al. 2014). Our findings are consistent with earlier research showing that the inclusion of hydrophobic substances increased the contact angle of the edible agar/maltodextrin films (Zhang et al. 2019). Further investigation revealed that the addition of castor oil to the pectin and gluten films enhanced their surface hydrophobicity (Siracusa et al. 2018; Kashyap, Sistla, and Mehraj 2023). However, the contact angle of the developed films is less than that of pure LDPE films, that is 98.6 as observed in the previous study (Aktas et al. 2023).

### 3.4 Thickness, Mechanical, and Barrier Properties

As mentioned in Table 3, the developed composite films were in the range of 0.062–0.095 mm, which qualifies the criteria for thin film as defined by the American Society for Testing and Materials (ASTM) (1985) (Hazirah, Isa, and Sarbon 2016; ASTM 1990). The addition of MOGR increased the thickness value from 0.062 ± 0.005 to 0.095 ± 0.006 mm, which is still comparatively less than the thickness of the LDPE‐based films of 0.15 and 0.7 mm (Szlachetka et al. 2021). This can be the result of the inclusion of MOGR changing the structural homogeneity. These findings are in line with previous results where the thickness of polylactic acid biodegradable films increased with the addition of mastic gum (Aydogdu Emir, Akgun, and Kirtil 2023).

The composite films showed TS and EAB within the range of 4.92–7.30 Mpa and 29.70%–32.41%. It was found that an increase in the concentration of MOGR tensile strength decreased significantly from 7.30 to 4.92 Mpa, whereas a slight decrease in EAB values (32.41%–29.70%) was also observed. The decrease in the tensile strength with an increase in the concentration of MOGR could be attributed to the weakening of intermolecular forces. This could be due to the development of heterogeneous material after the addition of MOGR, as the presence of the OH group and hydrophobic content in MOGR could have interfered with intermolecular forces of the polymeric matrix, resulting in a decrease in the film's stiffness and resistance to elastic force, and therefore, dropped tensile strength for the films.

As per the general rule, with an increase in TS, EAB values decreased in biopolymer‐based films (Tong, Xiao, and Lim 2008). However, findings obtained in our work presented a reduction in TS and EAB values. These findings were in accordance with the study where xanthan gum addition to gelatin‐carboxymethyl cellulose film reduced the TS and EAB values (Hazirah, Isa, and Sarbon 2016). The tensile strength values of the films studied were greater than the TS of polyethylene blank extruded films (18.2 MPa); however, the TS and EAB values of the studied films were less than the EAB (564%) and TS (120 MPa) of cellophane film (Ordon et al. 2021).

Table 3 displays the WVP values for all films. With an increase in MOGR concentration, the films' WVP increased significantly (*p* < 0.05) from 0.553 to 0.872 (g × mm)/(m^2^ × h × kPa). This could be due to the increased free volume in the films, allowing water molecules to diffuse more quickly. Similar results were observed when gum ghatti was incorporated into sodium alginate‐based films (Cheng et al. 2021). These results demonstrated that adding MOGR to films enhanced their water vapor permeability rather than improving their water vapor barrier. This mechanism can prevent condensation resulting from temperature gradients within the package and facilitate the diffusion of water vapor (evaporated from the product surface) through the film, thereby mitigating the accumulation of moisture inside the package (Linke and Geyer 2013).

**TABLE 3:** Thickness, mechanical and barrier properties mean values of the control and MOGR loaded film samples.

Film samples	Thickness (mm)	TS (MPa)	EAB (%)	WVP [(g × mm)/(m^2^ × h × kPa])
PCM/Control	0.062 ± 0.004^a^	7.30 ± 0.50^a^	32.41 ± 1.0^a^	0.553 ± 0.013^a^
PCM1	0.073 ± 0.005^b^	5.49 ± 0.43^b^	30.66 ± 0.55^b^	0.684 ± 0.016^b^
PCM2	0.088 ± 0.005^c^	5.22 ± 0.48^b^	30.62 ± 0.55^b^	0.776 ± 0.017^c^
PCM3	0.095 ± 0.006^c^	4.92 ± 0.34^b^	29.70 ± 0.24^c^	0.872 ± 0.012^d^

*Note:* The ± sign means standard deviations. Significant differences (*p* < 0.05) are denoted by distinct letters (a, b, c, and d) within a column.

### 3.5 Opacity and Color Attributes of the Films

Opacity and color assessment of the films helps in meeting end consumer requirements. Table 4 presents the opacity and color parameters of the pectin and sodium alginate blank and MOGR‐based films. The opacity of the films increased with the addition of MOGR to the fabricated films (Table 4). Furthermore, as indicated by Table 4, the addition of MOGR to the edible films made them more yellowish, and as a result, the b value increased. This might be because the MOGR contains flavonoids and phenolic acids. (Rahmani et al. 2022). Additionally, there was a significant (*p* < 0.05) increase in the films' ∆E value with an increase in MOGR concentration.

**TABLE 4:** Opacity and color parameters of the pectin and k‐carrageenan‐based fabricated films.

Sample codes	Opacity	*L*	a*	b*	Δ*E*
PCM/Control	1.24 ± 0.07^a^	96.34 ± 0.02^a^	0.01 ± 0.01^a^	0.73 ± 0.05^a^	0.77 ± 0.03^a^
PCM1	6.41 ± 0.10^b^	96.81 ± 0.28^a^	−1.47 ± 0.07^b^	11.57 ± 0.35^b^	4.01 ± 0.21^b^
PCM2	8.51 ± 0.12^c^	93.65 ± 0.20^b^	−1.75 ± 0.10^b^	14.98 ± 0.82^c^	4.09 ± 0.16^b^
PCM3	9.41 ± 0.24^d^	89.38 ± 0.73^c^	−1.68 ± 0.16^b^	25.64 ± 0.98^d^	5.09 ± 0.49^c^

*Note:* The ± sign means standard deviations. Significant differences (*p* < 0.05) are denoted by distinct letters (a, b, c, and d) within a column.

Abbreviations: *L*, lightness; a*, green‐red color; b*, blue‐yellow color; ΔE*, overall color variation.

These results are consistent with visual observations of the films, which, as Figure 1 illustrates, exhibit increasing opacity and yellowness with increasing MOGR content. Furthermore, similar observations were reported in the previous studies where the addition of the extracts impacted the optical properties of the film (Bhatia, Wasef, et al. 2023; Jutaporn, Suphitchaya, and Thawien 2011; Yuan et al. 2020; Zhang, Li, and Jiang 2020).

### 3.6 Antioxidant Properties of the Films

**FIGURE 5:**
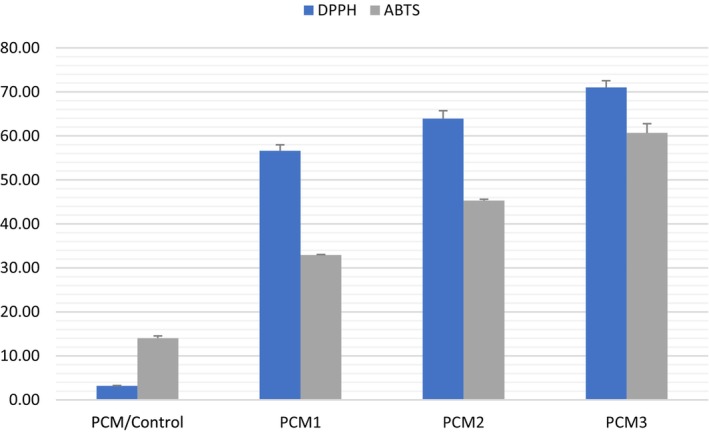
Antioxidant activities of the film samples including control and films loaded with MOGR. The error bars represent the standard deviation.

The antioxidant activity of blank and MOGR‐loaded composite films (25 mg) is shown in Figure 5. The DPPH and ABTS cation free radical scavenging activities of the blank composite films were 3.18% and 14.04%, respectively. The hydroxyl, sulfate, and carboxyl groups in the k‐carrageenan polymer chain may be the reason for higher antioxidant attributes (Sun et al. 2010). The present results are consistent with a prior investigation, which found that the blank carrageenan/alginate films exhibited free radical scavenging capabilities of 12.3% and 2.3% against ABTS and DPPH, respectively (Khan et al. 2023).

Additionally, the findings showed that as the MOGR content increased, the anti‐radical activity of the films improved significantly (*p* < 0.05) (Figure 5). PCM 3 films with the highest concentration of MOGR showed 71.03% for DPPH and 60.67% for ABTS. The greater levels of phenolic chemicals and flavonoids found in myrrh may be the reason for the improved antioxidant activity of the films (Khan et al. 2023). Myrrh, an oleo‐gum resin derived from a plant belonging to the genus Commiphora, contains resin, volatile oil, gum, and certain impurities. The alcoholic fraction of this oleo gum resin contains commiphorinic acids, commiferin, heerabomyrrhols, and heeraboresene. Triterpenes such as ursolic as well as oleanolic acid and essential oils in the resins of *C*. *myrrh* demonstrated potent antioxidant activity (Batiha et al. 2023). After research into the safety profile, the Food and Drug Administration (FDA) (1992) approved myrrh as a food additive, and it was subsequently added to the list of compounds that are generally regarded as safe (FDA 1992; Schrankel 2004; Suliman et al. 2022). For human consumption, myrrh is identified as harmless and certified as a natural flavoring agent by the US Food and Drug Administration (Ford, Letizia, and Api 1988). The higher antioxidant potential of the MOGR‐loaded film suggests its potential to control the oxidation of the foods.

### 3.7 X‐Ray Diffraction Analysis of the Films

**FIGURE 6:**
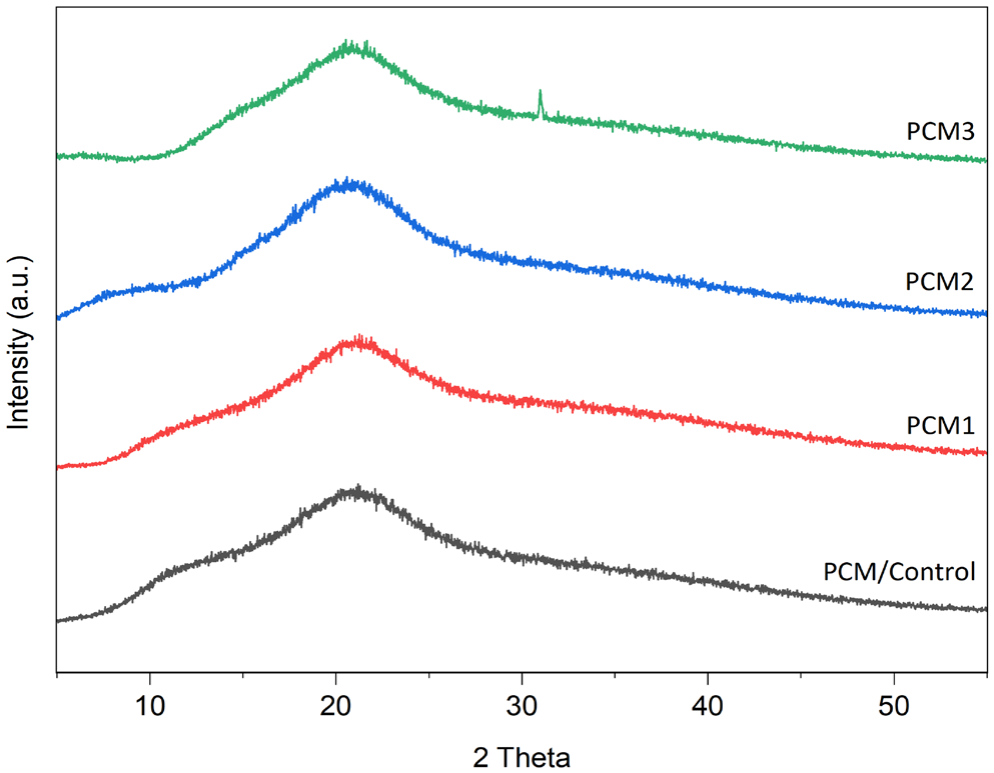
XRD analysis of the film samples including control and films loaded with MOGR.

Figure 6 illustrates the X‐ray diffraction (XRD) pattern of the composite films. Consistent with prior investigations, all films exhibited XRD profiles indicative of a semi‐crystalline arrangement, characterized by a prominent broad peak centered at 2*θ* = 21°. This finding suggests that the inclusion of a minor quantity of MOGR does not significantly alter the crystalline structure of the films (Adam et al. 2020). The presence of this peak indicates that the predominant phase of pectin and κ‐carrageenan is amorphous, with partial crystallinity. The principal broad peak at 2*θ* = 21 is likely attributable to κ‐carrageenan and pectin, as supported by earlier research (Adam et al. 2020; Azizi et al. 2017; Rhim and Wang 2014; Mendes et al. 2020). The possible cause of the distinctive peak observed at 2*θ* = 29.80° could be attributed to inorganic salts found in the κ‐carrageenan films, specifically potassium chloride (KCl) or the inorganic components present in the MOGR extract (Martins et al. 2012). Furthermore, the broadly defined peak reported around 12.7° (2*θ*) in the PCM blank sample could be related to the crystallinity of pectin (Nisar et al. 2018; Chaichi et al. 2017).

The addition of MOGR increased the crystallinity of composite edible films which could be due to the crosslinking effect of polyphenolic and flavonoid components present in MOGR increasing intermolecular interaction between polymers and MOGR. Similar results were observed in the previous study (Mondal et al. 2022). The enrichment of composite film with MOGR did not affect its amorphous character significantly at 21 (2*θ*). The incorporation of the MOGR did not impart any crystalline structure at this point to films, signifying that the possible interaction among polymers and the MOGR took place in the amorphous phase.

### 3.8 FTIR Analysis of the Films

**FIGURE 7:**
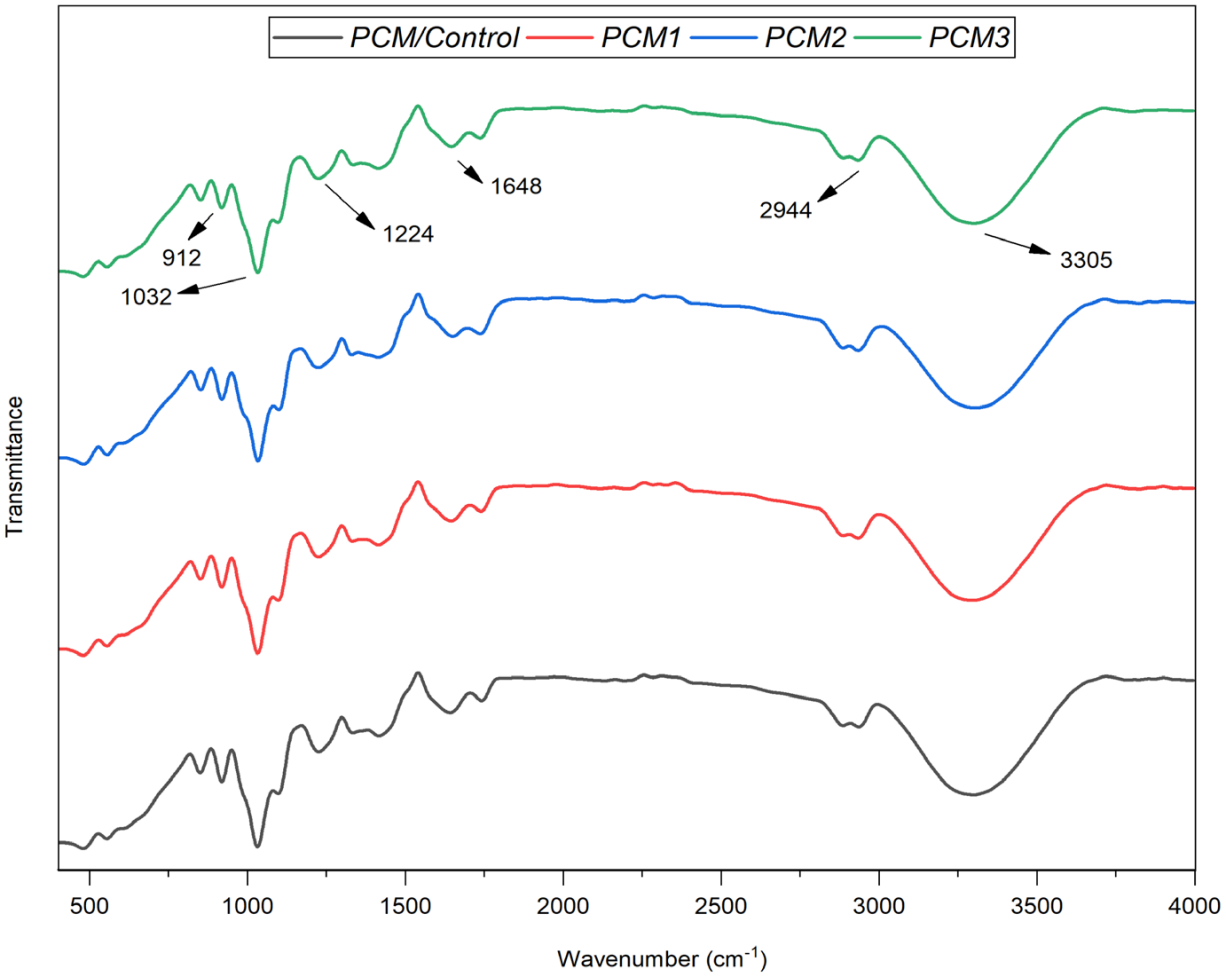
FTIR analysis of the film samples including control and films loaded with MOGR.

In all film samples, a broad band observed around 3305 cm^−1^ is attributed to the stretching vibration of O‐H and the presence of intramolecular and intermolecular hydrogen bonding, likely originating from constituents such as glycerol, pectin, and κ‐carrageenan (Figure 7) (Nisar et al. 2018; Khan et al. 2012). The peak at 2931 cm^−1^ corresponds to the vibration of –CH methylene groups within the polymer chains and methyl ester groups (Cabello et al. 2015). A band appearing at 1640 cm^−1^ in all samples indicates the bending vibration of O‐H bonds in absorbed water molecules. Additionally, the band near 1224 cm^−1^ may be associated with the ester sulfate group (O═S═O) present in κ‐carrageenan (Şen and Erboz 2010). The bands observed at 1032 cm^−1^ are likely related to –COC stretching vibrations within the film structure (Mujtaba et al. 2017). Furthermore, vibrations at 912 cm^−1^ are attributed to C–H bonds in β‐d‐glucopyranose units, while the band at 843 cm^−1^ may originate from O–SO₃ groups in galactose‐4‐sulfate. Comparison of spectra between blank films and those loaded with MOGR indicates no appearance of new bands, suggesting that the incorporation of MOGR did not result in any new chemical interactions between polymers and MOGR during film fabrication. This finding aligns with previous research indicating minimal spectral changes upon the addition of additives (Figure 7) (Distantina and Fahrurrozi 2013).

Overall, no new bands appeared in the films after MOGR was included, indicating that no new chemical interaction between polymers and MOGR occurred during the fabrication of the film, according to a comparison of the blank film's spectra with the films loaded with MOGR. Similar findings were observed in the previous study, where the incorporation of additives caused no significant changes in the spectrum (Gupta et al. 2021).

## 4 Conclusion

In conclusion, the incorporation of Myrrh oleo‐gum‐resin (MOGR) into pectin and K‐carrageenan composite films resulted in significant alterations in various physical, mechanical, and chemical properties. Despite these changes, the composite films demonstrated enhanced anti‐radical activity and reducing power, suggesting potential applications in functional packaging materials with added antioxidant properties. Overall, the study highlights the feasibility of utilizing MOGR as a natural additive in composite films, offering not only enhanced functional properties but also the potential for sustainable and eco‐friendly packaging solutions. Further research could explore optimization strategies to balance the conflicting effects on mechanical and barrier properties while maximizing antioxidant benefits. Additionally, investigating the release kinetics of bioactive compounds from these films during packaging applications would provide valuable insights into their efficacy for food preservation and shelf‐life extension.

## Author Contributions

**Yasir Abbas Shah:** conceptualization (equal), methodology (equal), software (equal), writing – original draft (equal), writing – review and editing (equal). **Saurabh Bhatia:** project administration (equal), supervision (equal), writing – original draft (equal), writing – review and editing (equal). **Sampath Chinnam:** formal analysis (equal), writing – review and editing (equal). **Ahmed Al‐Harrasi:** project administration (equal), supervision (equal). **Mohammad Tarahi:** formal analysis (equal), validation (equal), writing – original draft (equal). **Talha Shireen Khan:** data curation (equal), formal analysis (equal), visualization (equal), writing – review and editing (equal). **Tanveer Alam:** formal analysis (equal), methodology (equal), visualization (equal). **Esra Koca:** formal analysis (equal), methodology (equal), software (equal). **Levent Yurdaer Aydemir:** formal analysis (equal), methodology (equal), writing – review and editing (equal). **Anil K. Philip:** formal analysis (equal), writing – review and editing (equal). **Muhammad Afzaal:** data curation (equal), formal analysis (equal), writing – review and editing (equal). **Anubhav Pratap‐Singh:** formal analysis (equal), writing – review and editing (equal). **Mahbubur Rahman Khan:** formal analysis (equal), investigation (equal), writing – review and editing (equal).

## Consent

The authors have nothing to report.

## Conflicts of Interest

The authors declare no conflicts of interest.

## Data Availability

Even though adequate data has been given in the form of tables and figures, however, all authors declare that if more data is required then the data will be provided on a request basis.
